# Statistical optimization, characterization, antioxidant and antibacterial properties of silver nanoparticle biosynthesized by saw palmetto seed phenolic extract

**DOI:** 10.1038/s41598-023-42675-0

**Published:** 2023-09-20

**Authors:** Azza M. Abdel-Aty, Amal Z. Barakat, Roqaya I. Bassuiny, Saleh A. Mohamed

**Affiliations:** Molecular Biology Department, National Research Centre, Dokki, Cairo, Egypt

**Keywords:** Biochemistry, Biological techniques

## Abstract

On the global market, silver nanoparticles (Ag-NPs) are in high demand for their various applications in biomedicine, material engineering, and consumer products. This study highlighted the biosynthesis of the Ag-NPs using saw palmetto seed phenolic extract (SPS-phenolic extract), which contained vital antioxidant-phenolic compounds. Herein, central composite statistical design, response surface methodology, and sixteen runs were conducted to optimize Ag-NPs biosynthesis conditions for maximizing the production of Ag-NPs and their phenolic content. The best-produced SPS-Ag-NPs showed a surface plasmon resonance peak at 460 nm and nano-spherical sizes ranging from 11.17 to 38.32 nm using the UV spectrum analysis and TEM images, respectively. The produced SPS-Ag-NPs displayed a high negative zeta-potential value (− 32.8 mV) demonstrating their high stability. The FTIR analysis demonstrated that SPS-phenolic compounds were involved in sliver bio-reduction and in stabilizing, capping, and preventing Ag-NP aggregation. The thermogravimetric investigation revealed that the produced SPS-Ag-NPs have remarkable thermal stability. The produced SPS-Ag-NP exceeded total antioxidant activity (13.8 µmol Trolox equivalent) more than the SPS-phenolic extract (12.0 µmol Trolox equivalent). The biosynthesized SPS-Ag-NPs exhibited noticeably better antibacterial activity against multidrug-resistant Gram-negative *E. coli* and Gram-positive *S. aureus* compared to SPS-phenolic extract. Hence, the bio-synthesized SPS-Ag-NPs demonstrated great potential for use in biomedical and antimicrobial applications.

## Introduction

Nanotechnology is a rapidly developing field of science with many applications in the food industry, food safety, healthcare, biomedicine, pharmaceutical industry, environment, water treatment, and cosmetics^1–3^. Nanoparticles (NPs), particles size less than 100 nm, are one of the great findings of nanotechnology to address the difficulties/problems that face the current world^4^. NPs possess distinctive structures and improved properties due to their very small sizes and larger surface area compared to their volume ratio and could be employed in a variety of design materials with unique features^5^. Nowadays, the production of metal-NPs with the best possible physical and chemical properties is the main target for many scientists. However, the production of metal-NPs through chemical processes, microwave irradiation, or thermal degradation involved harmful waste products. Therefore, natural mechanisms including bacterial, fungal, and plant extracts are the best way to generate safe, clean, and biocompatible metal-NPs^6^.

Silver nanoparticles (Ag-NPs) have sparked significant interest due to their unique physical, chemical, and optical properties and are implicated in many applications including drug delivery, biological detection, catalysis, antimicrobial, and wound healing^7–9^. Ag-NPs are well established in the cosmeceutical industry due to their broad spectrum of pharmacology applications^10^. The global Ag-NPs market has grown significantly from 1.1 billion USD in 2016 to 3.0 billion USD by 2021. Ag-NPs could be produced via many chemical and physical methods such as chemical reducing agents, stabilizers, or capping materials. Unfortunately, these methods are expensive and need many hazardous chemical reagents and complex steps. The bio/green synthesis of Ag-NPs is a safe, clean, and rapid technology that could minimize the impact of toxic chemicals on human health and the environment. Some reports use natural materials like plant extracts and microbial enzymes for the biosynthesis of Ag-NPs^11,12^. Plant metabolites converted Ag ions into Ag metals and stabilized the size and shape of the generated Ag-NPs. In the green synthesis of Ag-NPs, various plant metabolites including proteins, carbohydrates, terpenoids, and alkaloids have been reported^13^. However, phenolic compounds are the main plant metabolites implicated in this process because of their potent reducing properties and the great stability of biosynthesized Ag-NPs^14,15^.

Saw palmetto (*Serenoa repens*, family Arecaceae) is a dwarf palm native to North America and is commonly planted in Egypt. Saw palmetto berry extract (SPE) is the most common herbal supplement and has several pharmacological activities and is clinically effective in treating urological diseases. The SPE extract mainly contains free fatty acids and sterols, with minor amounts of fatty alcohols, flavonoids, and polyphenols^16^. On the contrary, the saw palmetto seeds phenolic extract (SPS) had a substantial amount of advantageous antioxidant-phenolic compounds with antibacterial, anti-inflammatory, and anti-diabetic activities. The majority of the SPS-phenolic extract is composed of antioxidant-phenolic acids such as protocatechuic, gallic, caffeic, *p*-hydroxybenzoic, syringic, and chlorogenic acids^17,18^. These phenolic acids are potent chelating and/or reducing agents that could effectively convert metal ions to metal-NPs^19^. Therefore, this study attempts to assess the potential of SPS-phenolic compounds/extract as a reducing and capping agent for bio-synthesis Ag-NPs using response surface methodology for optimizing the production conditions. In addition, the structural, morphological, thermal stability, antibacterial, and antioxidant properties of the generated Ag-NPs were investigated.

## Materials and methods

### Plant material

Saw palmetto seeds were provided by Giza Agriculture Research Centre (ARC). The fully grown saw palmetto fruits were collected in October 2021 and identified by the Botany Department at ARC in Giza, Egypt, with the number 810-2021#. The seeds were removed from saw palmetto fruits after one day-harvesting.

### Extraction of phenolic compounds from Saw palmetto seeds

The phenolic compounds of the Saw palmetto seed (SPS) were extracted according to the method of Barakat et al.^17^ with minor modifications. The SPS were cleaned, dried at 45 °C in the oven, and ground. Ten g of powdered SPS were shaken with 100 ml of 80% methanol at 200 rpm and at room temperature for 24 h. The produced extract was filtered via Whatman-1 filter paper and designated as an SPS-phenolic extract.

### Ag-NPs biosynthesis-optimization conditions and statistical design

**Figure 1:**
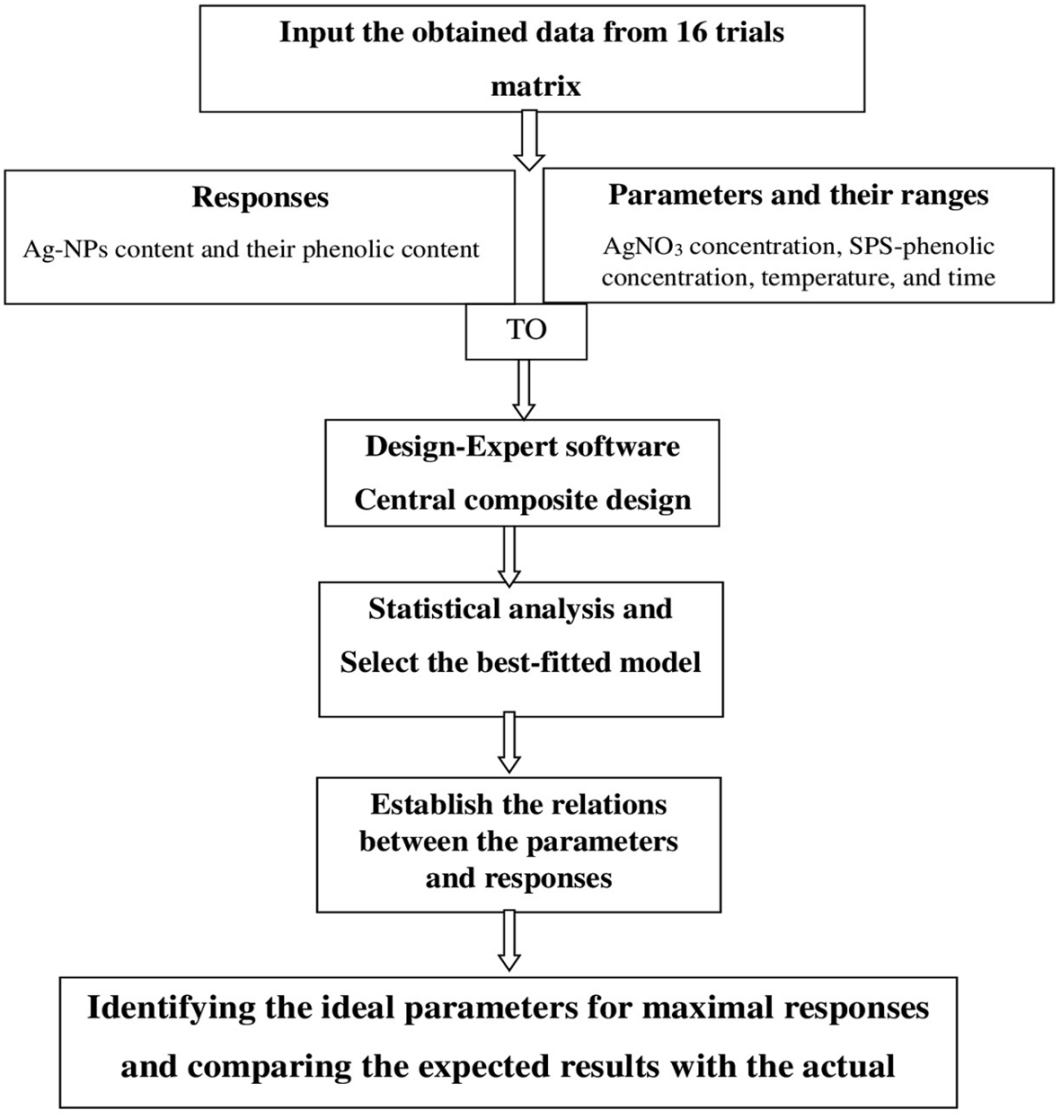
The statistical design software steps for optimization of the Ag-NPs biosynthesis conditions and for maximum yield production.

Many trials and single-parameter experiments were preliminary performed for the selection of the best Ag-NPs biosynthesis conditions. Central composite design (CCD) and response surface methodology (RSM) of Design Expert^®^ software version 11 and sixteen trials/experiments were applied to statistically optimize Ag-NPs biosynthesis conditions. The sixteen different nanoparticle formulations were conducted at 4 independents variables: SPS-phenolic extract concentrations (2.5 and 5.0 mg gallic acid equivalent), AgNO_3_ concentrations (20 and 40 mM), temperatures (30 and 60 °C), and incubation times (1 and 2 h). The obtained bio-reduced Ag-NPs solution was freeze-dried at − 55 °C for 24 h. The weight (mg) and the TPC (mg gallic acid equivalent) for the obtained Ag-NPs were measured to assess the best conditions of the Ag-NPs biosynthesis. Figure 1 screens the statistical design software steps used in the optimization of silver nanoparticle production.

### Determination of phenolic content

By using the Folin-Ciocalteure reagent and according to the method of Velioglu et al.^20^ the total phenolic content (TPC) of the SPS-phenolic extract was assayed. The TPC was calculated as mg gallic acid equivalent (mg GAE)/gram seeds.

### Antioxidant activity

The antioxidant properties of the SPS-phenolic extract and biosynthesized-Ag-NPs were tested using the 1,1-Diphenyl-2-picrylhydrazyl (DPPH) assay^21^. After mixing 100 µl of the extract with 100 µl of DPPH reagent and 800 µl of methanol and incubating in the dark for 30 min, the absorbance was then read at 517 nm.

### Antibacterial activity

*Escherichia coli* O157-H7 ATCC 51659 (Gram-negative bacterial-strain) and *Staphylococcus aureus* ATCC 13565 (Gram-positive bacterial-strain) were obtained from the Food-Toxins and Contaminants Department, NRC, Cairo, Egypt, and were used to evaluate the antibacterial efficiency of both the SPS-phenolic extract and biosynthesized-Ag-NPs. Briefly, 100 µl of each bacterial suspension, which contained 10^8^ CFU/ml, was applied to the agar plate. The samples, each at a concentration of 50 µg/200 µl, were added to prepared wells in the agar plates, and incubation take place for 18 ± 1.0 h at 37 ± 1.0 °C. Antibacterial activity was measured using the inhibitory zone diameters. To test the minimum inhibition concentration (MIC) of the SPS-extract and biosynthesis-Ag-NPs, different concentrations of both were added to contaminant agar plates, and the incubation was conducted under the above-mentioned conditions. The MIC is the lowest concentration at which bacteria cannot grow.

### Characterization of biosynthesized Ag-NPs

#### UV spectrum analysis

The UV spectrum analysis of the prepared/biosynthesized Ag-NPs ranging from 300 to 800 nm was recorded via a UV–Visible spectrophotometer at room temperature (30 °C).

#### Morphology analysis of the biosynthesized Ag-NPs

The prepared/biosynthesized Ag-NPs surface morphology was imaged via Transmission Electron Microscopy (TEM) (JEOLJEM-1200, Japan) at 80 kV to identify the particle agglomeration and size distribution.

#### Zeta potential (ZP) and dynamic light scattering (DLS)

The average diameter, size distribution, and zeta potential for the prepared/biosynthesized Ag-NPs were measured using a particle size analyzer (Nano-ZS, Malvern Instruments Ltd., UK). Ten mg of Ag-NPs were suspended in 5 ml of saline, filtered, and sonicated for 10–20 min just before analysis. Malvern instrument dispersion technology software was used.

#### FTIR-analysis

To analyze/investigate the functional groups of the prepared/biosynthesized Ag-NPs, the Fourier Transform Infrared (FT-IR) technique was employed (Bruker ALPHA-FTIR-Spectrometer), with platinum-attenuated reflection waves ranging from 400 to 4000 cm^1^.

#### Thermal analysis

To evaluate the thermal properties of the prepared biosynthesized Ag-NPs, thermogravimetric analysis (TGA) and differential thermogravimetric (DTG) experiments were conducted. Runs were carried out between 40 and 800 °C at a constant heat rate (10 °C/min).

All experimental procedures were carried out in compliance with relevant guidelines.

### Statistical analysis

The biosynthesis Ag-NPs-physiological conditions were optimized using CCD and RSM of Design Expert® software version 11. A matrix of 16 runs was conducted, and the statistical results, model design, and equation were all validated using ANOVA with a *p*-value of less than 0.05. The remaining data were examined with a one-way ANOVA followed by a Tukey post-test (Graph Pad Prism 5 software). All values were presented as means ± SD (n = 3).

## Results and discussion

### Ag-NP biosynthesis optimized formulation based on the design expert

**Figure 2:**
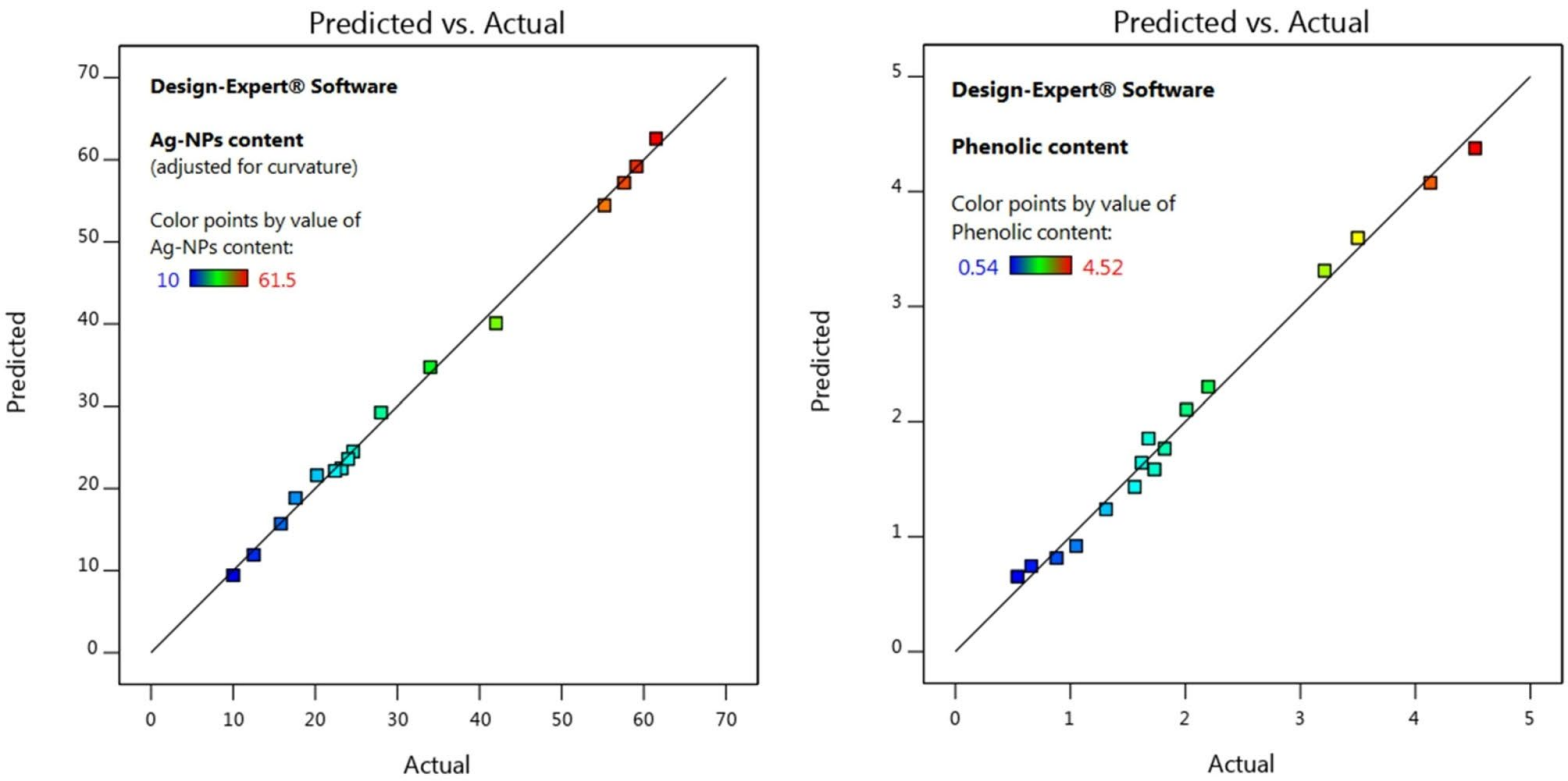
Scatter graphs of predicted values vs actual values for obtained Ag-NPs formulations and their phenolic contents.

Setting/optimizing NP biosynthesis conditions is a very challenging process since several variables (such as temperatures, pHs, times, and concentrations) interact simultaneously. So, many multivariance-analytical models were conducted to adjust the process conditions^16,22,23^. Utilizing statistics can be able to address these difficulties and also potentially reduce the cost and time required to complete the task^24^. Herein, the statistical design expert software was employed to investigate the best conditions for SPS-Ag-NP formation. Four empirical synthesis variables Ag^+^ (A) and phenolic concentrations (B), temperature (C), and time (D), and their effects on the obtained Ag-NPs formulation and total phenolic content. Table 1 analyzes the actual and predicted Ag-NPs obtained formulations from sixteen trials to determine the ideal Ag-NPs green synthesis conditions for producing the highest yield of Ag-NPs content (mg) and their phenolic content (GAE mg). In addition, Fig. 2 shows that the actual and predicted data were bilaterally scattered and extremely near to one another, demonstrating the high precision of the predicted and experimental data for both Ag-NPs and their phenolic contents. The findings demonstrated a strong correlation between the rise in silver nitrate concentration, phenolic extract concentration, temperature, and incubation time and the increase in Ag-NPs yield and their phenolic content. Silver nitrate and phenolic extract concentrations as well as the temperature have a significant/positive impact on the generation, shape, size, and size distribution of Ag-NPs^25–27^.

**Table 1:** Screening of the experimental, predicted, and residual values for Ag-NPs obtained formulations and their phenolic contents under 16 trials/experiments.

Run	Ag (mg/20 ml)	SPS-concentration (mg GAE)	Temperature (°C)	Time (h)	Ag-NPs content (mg)		Residual	Phenolic content (mg GAE)		Residual
					Actual	Predicted		Actual	Predicted	
1	40	2.5	30	1	10.00	9.42	0.5813	0.5400	0.6537	− 0.1137
2	40	2.5	30	2	12.50	11.92	0.5813	0.6600	0.7437	− 0.0837
3	40	2.5	60	1	15.80	15.72	0.0812	0.8800	0.8137	0.0663
4	40	2.5	60	2	17.60	18.84	− 1.24	1.05	0.9187	0.1313
5	40	5.0	30	1	20.20	21.62	− 1.42	1.31	1.24	0.0713
6	40	5.0	30	2	22.40	22.14	0.2563	1.56	1.43	0.1263
7	40	5.0	60	1	23.20	22.44	0.7563	1.62	1.64	− 0.0237
8	40	5.0	60	2	24.00	23.59	0.4063	1.68	1.85	− 0.1737
9	86.5	2.5	30	1	24.60	24.52	0.0813	1.73	1.59	0.1438
10	86.5	2.5	30	2	28.00	29.24	− 1.24	1.82	1.77	0.0538
11	86.5	2.5	60	1	34.00	34.74	− 0.7437	2.01	2.11	− 0.0962
12	86.5	2.5	60	2	42.00	40.09	1.91	2.20	2.30	− 0.1012
13	86.5	5.0	30	1	55.20	54.44	0.7562	3.21	3.31	− 0.1012
14	86.5	5.0	30	2	57.60	57.19	0.4062	3.50	3.60	− 0.0962
15	86.5	5.0	60	1	59.10	59.19	− 0.0938	4.13	4.08	0.0537
16	86.5	5.0	60	2	61.50	62.57	− 1.07	4.52	4.38	0.1437

SPS: Saw palmetto seed phenolic extract; GAE: Gallic acid equivalent; Ag-NPs: Sliver nanoparticles biosynthesized using saw palmetto seed phenolic extract. (40 mg/20 ml) and (86.5 mg/20 ml) equivalent to the sliver weight at 20 and 40 mM, respectively.

A statistically fitted model and ANOVA variance analysis were used to verify the values of the actual and predicted yield of Ag-NPs contents and their phenolic levels. This analysis suggested that the two-factor interaction (2FI) model is the best-fitted model for these responses (Ag-NPs contents and their phenolic levels), achieving very significant P-values and F-values as shown in Table 2. Moreover, the predicted R_2_ values for Ag-NP and their phenolic content of 0.9937 and 0.9878, respectively, were harmonized with the adjusted R_2_ values of 0.9960 and 0.9808; respectively. A sufficient signal-to-noise ratio was also shown by greater adequate precision values of 82.52 and 48.56 for the Ag-NPs formulation and their phenolic contents, respectively (Table 2). Finally, this analysis indicates the precision and accuracy of the suggested model.

**Table 2:** ANOVA analysis and fit statistics for 2FI models.

ANOVA				Fit statistics for model			
Responses	F-value	*p*-value	Model	R^2^	Adjusted R^2^	Predicted R^2^	Adeq precision*
Ag-NPs content	773.19	< 0.0001	2FI	0.9973	0.9960	0.9937	82.52
Phenolic content	252.23	< 0.0001	2FI	0.9917	0.9808	0.9878	48.56

*The Adeq. precision displays the signal-to-noise ratio. A value of more than 4 is better.

**Figure 3:**
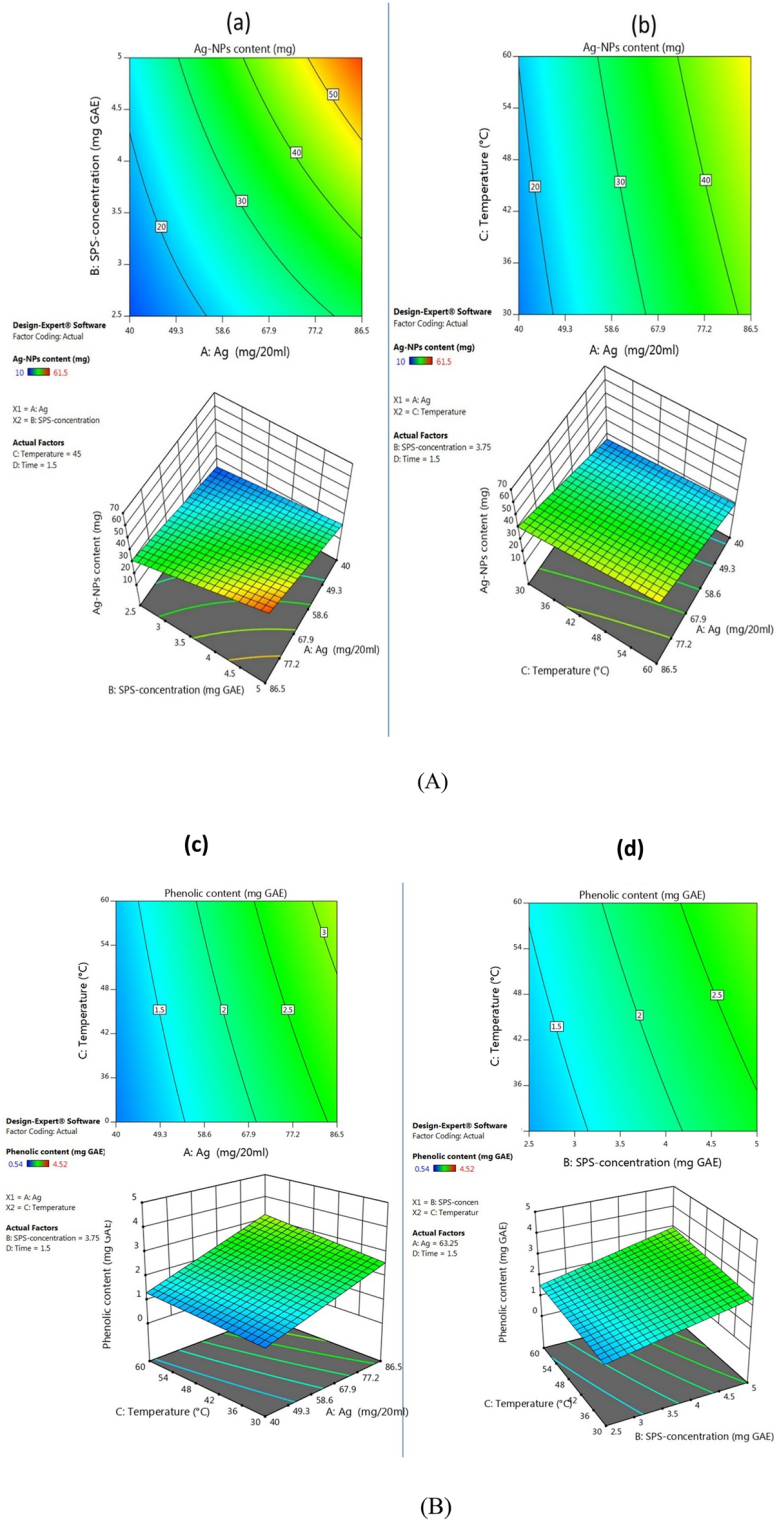
(**A**) Contour and 3D surface graphs of the Ag-NPs biosynthesis conditions and their interactions for the maximum producing Ag-NPs content. Ag^+^ and SPS concentrations (a); and Ag^+^ concentration and temperature (b). (**B**) Contour and 3D surface graphs of the Ag-NPs biosynthesis conditions and their interactions for the maximum producing Ag-NPs-phenolic content. Ag^+^ and temperature (c); and SPS-concentration and temperature (d).

The influence of the examined Ag-NPs biosynthesis conditions (A, B, C, and D) and their reciprocal interactions (AB, AC, AD, BC, BD, and CD) on the Ag-NPs yields and their phenolic contents are seen in Fig. 3A and B and Table 3. The P-values of the Ag-NPs biosynthesis conditions (A, B, C, D) for both responses were less than 0.0001 indicating the significance of the suggested model and the strong impact of all the examined conditions and their ranges on the responses (Ag-NPs yields and their phenolic contents). The central composite statistical model used to optimize Ag-NPs biosynthesized by green tea extract demonstrated that the responses were significant for all the analyzed parameters^28^. On the contrary, the Box-Behnken statistical model used to optimize Ag-NPs biosynthesized by pomegranate leaf extract was found to be not significant due to the tested conditions with ranges that should be adjusted, less or more^14^. The 3D and contour charts are used to show the interacting effect of the parameters and responses. Those plots showed a function of two parameters at a time, holding all other parameters fixed at their center, to examine their impacts (Fig. 3A and B). The interactions between the terms AB, AC, and BC were positively significant for both responses. However, AD and BD were positively significant for only Ag-NPs content (Table 3). The following equations are the final equations that demonstrate the relations between the significant Ag-NPs biosynthesis conditions and their interactions and responses obtained from the suggested model, including their real effects:

$$\begin{aligned} {\text{Ag}} - {\text{NPs content}} &= + {31}.{73} + {13}.{\text{52 A }} + {8}.{\text{67 B }} + {2}.{\text{92 C}} \hfill \\ & \quad + {1}.{\text{47 D }} + {4}.{\text{43 AB }} + \, 0.{\text{9812 AC }} + 0.{\text{5563 AD }} \hfill \\ & \quad - { 1}.{\text{18 BC }} - 0.{\text{3833 BD }} + \, 0.0{\text{167CD}}. \hfill \\ \end{aligned}$$

$$\begin{aligned} {\text{Phenolic content}} & = + {2}.0{3} + \, 0.{\text{8637A}} + \, 0.{665}0{\text{ B }} + \, 0.{235}0{\text{C}} \hfill \\ & \quad + \, 0.0{\text{975 D }} + \, 0.{285}0{\text{ AB }} + \, 0.0{9}00{\text{AC}} + \, 0.0{\text{225 AD }} \hfill \\ & \quad + 0.0{\text{283 BC }} + \, 0.0{\text{183 BD }} - 0.00{\text{33 CD}}. \hfill \\ \end{aligned}$$

**Table 3:** Regression coefficients for Ag-NPs-biosynthesis conditions and their interactions.

	Intercept	A	B	C	D	AB	AC	AD	BC	BD	CD
Ag-NPs content	31.7312	13.5188	8.66875	2.91875	1.46875	4.43125	0.98125	0.55625	1.36875	0.49375	0.15625
p-values	–	< 0.0001*	< 0.0001*	< 0.0001*	< 0.0001*	< 0.0001*	< 0.0001*	0.0093*	< 0.0001*	0.0190*	0.4301
Phenolic content	2.02625	0.86375	0.665	0.235	0.0975	0.285	0.09	0.0225	0.06125	0.02625	0.00375
p-values	–	< 0.0001*	< 0.0001*	< 0.0001*	0.0004*	< 0.0001*	0.0008*	0.3414	0.0150*	0.2688	0.8727

Ag^+^ concentration (A), SPS-phenolic extract concentrations (B), temperature (C), and incubation time (D).

*Significant (< 0.0001–< 0.05).

**Figure 4:**
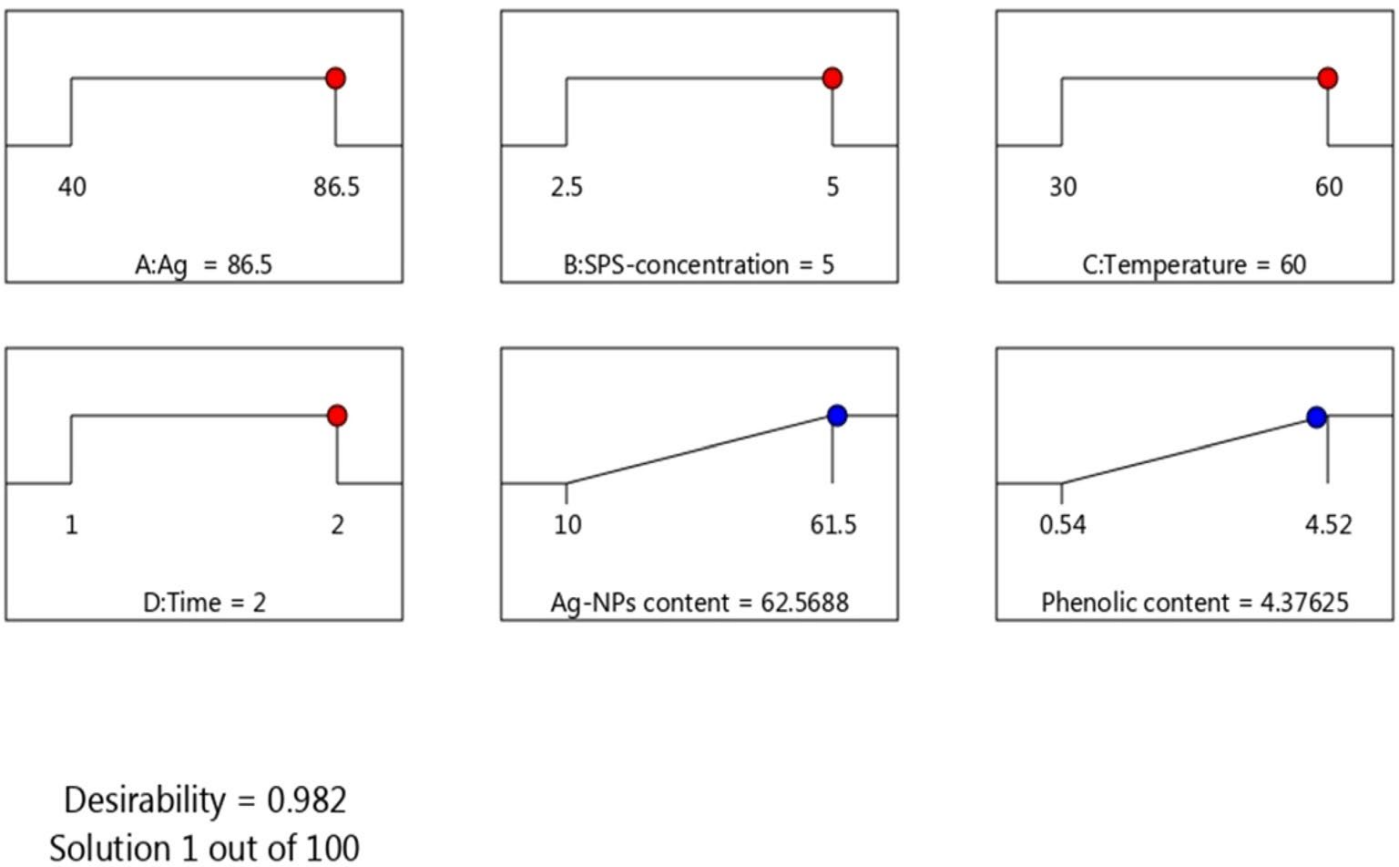
Desirability plots for the ideal Ag-NPs biosynthesis conditions with the highest yields possible for the Ag-NPs content and phenolic content.

Figure 4 shows the optimal conditions using a desirability ramp analysis for maximizing the production of Ag-NPs content (mg) and their phenolic content (mg GAE). The criteria in the numerical optimization were set in range for all conditions and maximizing the response index. The analysis shows that the conditions of Ag^+^-concentration 86.5 mg, SPS-phenolic concentration 5 mg GAE, temperature 60 °C and time 2 h provide the expected maximum Ag-NPs content and their phenolic content of 62.56 mg and 4.37 mg GAE, respectively, compatible with the experimental values of 61.5 mg and 4.52 mg GAE, respectively. Additionally, the desirability value of 0.982 showed that this solution met more than 98.2% of the maximized targets (Fig. 4). This demonstrates the model's applicability and validity. Overall, the optimal Ag-NPs biosynthesis conditions have been demonstrated to be 5.0 mg GAE of SPS-phenolic extract, 40 mM of AgNO_3_, 60 °C, and a 2-h incubation time for the highest production of SPS-Ag-NPs. The statistical experimental designs were successfully used for process condition optimization in several fields, such as pharmaceutical product development, food bioprocessing, fermentation, enzyme production, and many other industries^22,23,29^. However, there were few previous studies that used statistical designs to optimize Ag-NPs biosynthesized by plant extract. The Box-Behnken design and fourteen runs were conducted at 3 independent variables (temperature, silver nitrate concentration, and extract concentration), and Ag-NPs particle size and polydispersity index were analyzed as responses and recorded optimal Ag-NPs biosynthesis conditions at 0.83 mg/ml pomegranate leaf-phenolic extract, 1.15 mM AgNO_3,_ and 67 °C^14^. While the central composite design showed ideal Ag-NPs biosynthesis conditions at 1 mM AgNO_3_, 0.5% green tea extract, and 80 °C^28^. These differences in the Ag-NPs synthesis optimal conditions may be due to differences in parameter ranges, plant extracts, statistical models, and the measured responses.

### UV spectrum analysis

**Figure 5:**
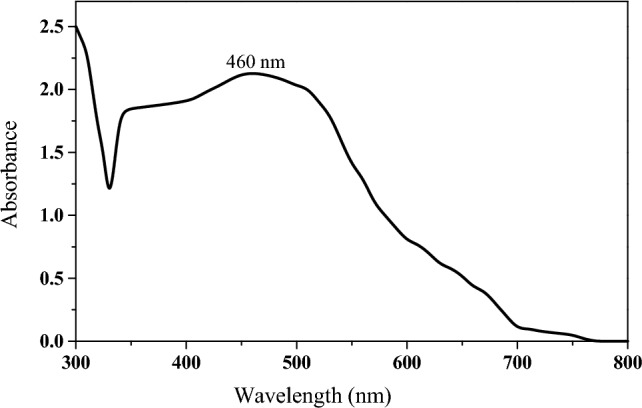
UV–Vis absorption spectrum of the Ag-NPs biosynthesized by SPS-phenolic extract, displaying the characteristic surface plasmon resonance band at 460 nm.

The prepared SPS-Ag-NPs were initially recognized visually by observing the color changes in the reaction solutions. These changes were connected to the stimulation of surface plasmon resonance (SPR) in the metal-NPs. Metal-NPs display an SPR absorption band due to their free electrons-vibration combined with the light wave^30^. UV-spectroscopy is an accurate technique for monitoring Ag-NPs synthesis by detection of the SPR absorption band. The development of the broad intense peak at 460 nm under the selected optimal conditions indicates the formation of Ag-NPs, as seen in Fig. 5. This single SPR peak suggests that the prepared SPS-Ag-NPs have spherical shapes^31^.

### Morphology of biosynthesized Ag-NPs

**Figure 6:**
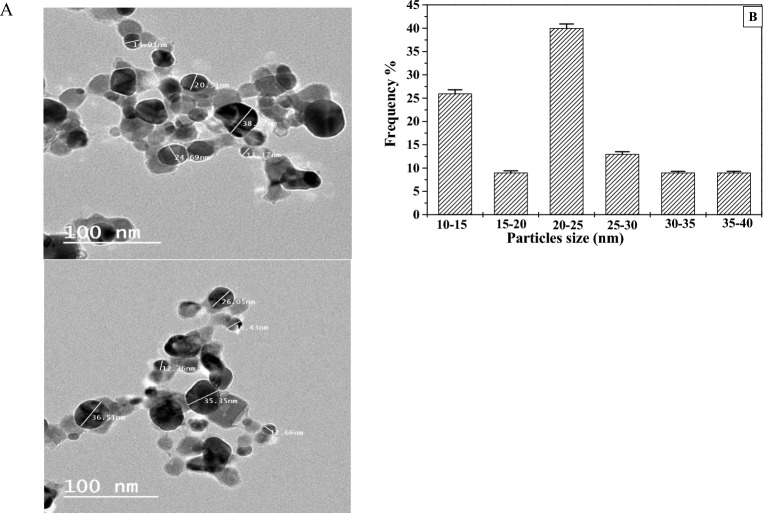
(**A**) TEM images of the obtained Ag-NPs biosynthesis by SPS-phenolic extract and (**B**) the histogram of particle size distribution.

TEM images of the biosynthesized Ag-NPs using the SPS-phenolic extract were presented in Fig. 6A. TEM morphology analysis showed spherical Ag-NPs with narrow particle diameters varying from 11.17 to 38.32 nm. In addition, the biosynthesized-Ag-NPs black particles were dispersed in gray outer regions, indicating that the abundant phenolic compounds of the SPS-phenolic extract capped the obtained Ag-NPs and prevented contact/agglomeration between the formed Ag-NPs. Likewise, some studies reported that the Ag-NPs were covered by the phenolic compounds when biosynthesized via plant extracts and they showed grey outer rings in TEM images^19,32,33^. Moreover, the histogram of the prepared Ag-NPs particle size distribution displayed that the most distributed particle sizes were in the range of 20–25 nm, followed by 10–15 nm with distribution percentages of 40 and 25%, respectively, as seen in Fig. 6B. Most of the biosynthesized-Ag-NPs using phenolic-rich extracts showed relatively smaller particle sizes > 35 nm than those obtained from crude aqueous extracts (35–70 nm)^14,34,35^.

### DLS and ZP

**Figure 7:**
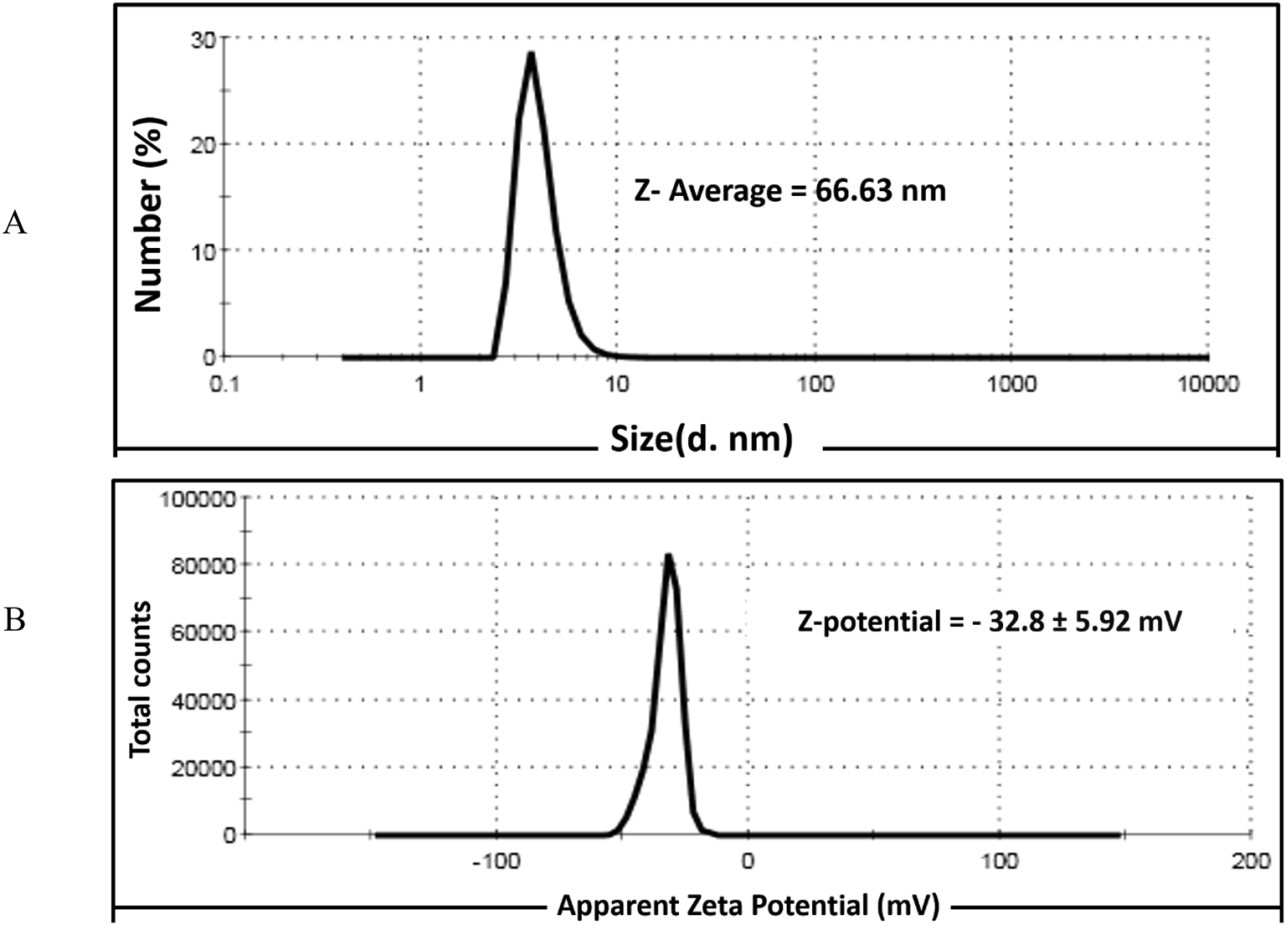
(**A**) Dynamic light scattering (DLS) and (**B**) Zeta potential (ZP) of the biosynthesized Ag-NPs by SPS-phenolic extract.

The DLS technique examines the shell thickness of capping material and hydrodynamic size of the generated Ag-NPs, as contrasted with TEM, which only measures the metallic core. The average size of the formed SPS-Ag-NPs was 66.63 nm as presented in Fig. 7A. The DLS-derived size (66.63 nm) was larger than that obtained by TEM (11.17 to 38.32 nm). This is because the DLS-measured size additionally includes SPS-phenolic compounds that capped the formed Ag-NPs and surrounded their core. According to the method used to assess particle size, Ag-NP sizes increased in the following order: DLS > TEM > XRD^36,37^. Furthermore, large size may also result from the interaction of several forces inside the solution.

Generally, the ZP data provide details on the stability and surface charge of synthesized Ag-NPs. The ZP value of the formed SPS-Ag-NPs was − 32.8 ± 5.92 mV (Fig. 7B). This negative ZP value demonstrated the high stability of the biosynthesized Ag-NPs^38^. The negatively charged phenolic compounds that cover the surface of the bio-formed Ag-NPs may be the cause of the negative ZP value^39^. The ZP measurement also confirms the interaction between the organic matrix (SPS-phenolic compounds) and the silver nanoparticles^40^.

### FTIR-spectra analysis

**Figure 8:**
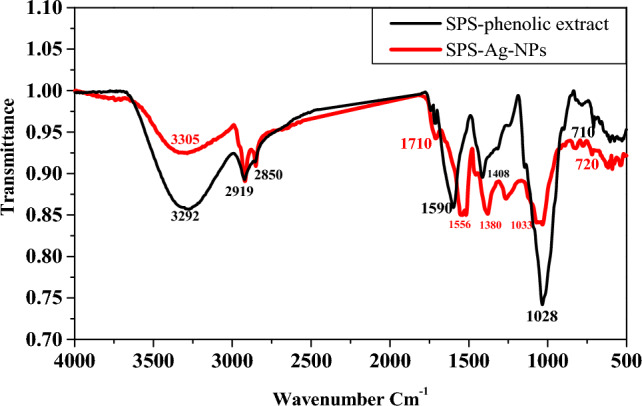
FT-IR analysis of the SPS-phenolic extract and biosynthesized SPS-Ag-NPs.

As can be seen in Fig. 8, the FTIR spectrum of the SPS-phenolic extract showed the main functional groups of the phenolic compounds. The functional peak at 3292 cm^−1^ was attributed to several O–H groups stretching vibrations, and the peaks at 2919 and 2850 cm^−1^ were attributed to C–H stretching vibrations. Further, the C=C stretching vibrations at 1590 cm^−1^, the −C−H bending vibrations at 1408 cm^−1^, C−O−C stretching vibrations at 1028 cm^−1^, and aromatic groups at 710 cm^−1^, all the identified peaks/groups are characteristic of the phenolic compound structures. Following the green biosynthesis process the prepared SPS-Ag-NPs spectrum recognized most functional groups/absorbed bands of SPS-phenolic compounds with minor shifting and different intensities. The O–H, C=C, −C−H, C−O−C, and C≡C groups were shifted from 3292, 1590, 1408, 1028, and 710 to 3305, 1556, 1380, 1033, and 720 cm^−1^, respectively. Interestingly, a strong peak around 1710 cm^−1^ corresponded to carbonyl groups (C=O) implicated in nanoparticle formation^41^. These investigations demonstrate the involvement of SPS-polyphenols in the bio-synthesized Ag-NPs. In addition, the SPS-phenolic extract is rich in many phenolic acids such as protocatechuic, gallic, chlorogenic, caffeic, and syringic as well as catechin as a flavonoid^17^, and their identified functional groups are implicated in the bio-reduction of the Ag ions into Ag-NPs and in stabilization, capping, and prevention from aggregation. Most relevant studies concluded that phenolic acids are the major bioactive compounds implicated in Ag-NPs biosynthesis^19,42,43^. The hydroxyl and carbonyl groups of phenolic acids can inactivate silver ions through chelation. The potent chelating activity of phenolic acids is probably due to the high nucleophilicity (high electron release ability) of their aromatic rings. Phenolic acids release electrons that can convert Ag^+^ to Ag and generate phenolic free radical derivatives. These derivatives reduce other Ag^+^ and are oxidized to o-quinones. These quinones coupled the produced Ag-NPs to create a steric barrier around them preventing Ag-NPs from aggregating and stabilizing their dispersion. This possible mechanism is suggested for the biosynthesis of Ag-NPs using SPS-phenolic extract based on some previous reports integrated with our FTIR analysis findings^19,44,45^.

### Thermal gravimetric analysis

**Figure 9:**
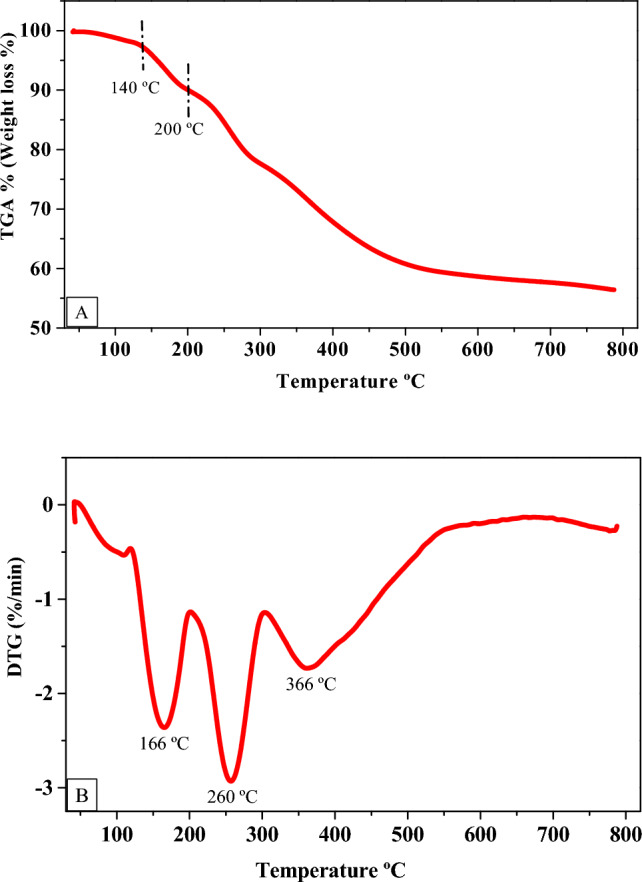
TGA curve (**A**) and DTG curve (**B**) of the biosynthesized Ag-NPs by SPS-phenolic extract.

The capping Ag-NPs biosynthesized by SPS-phenolic extract was further characterized using TGA and DTG thermal analyses. The TGA plot (Fig. 9A) shows two-step thermal decomposition. A late start of initial decomposition with a mass loss of 10% between 140 and 200 °C is due to the evaporation/dehydration of absorbed water. And then a steady mass loss appeared between 200 and 800 °C, reaching to maximized mass loss of 42%. This loss may be caused due to the decomposition of the phenolic compounds that capped the prepared Ag-NPs, demonstrating that the surface of the prepared Ag-NPs was altered/modified by SPS-phenolic compounds and supporting the FTIR findings. The thermal decomposition of the phenolic compounds might cause mass loss for Ag-NPs that were bio-synthesized by some plant extracts^46,47^. In the DTG plot, the highest decomposition rate of the prepared Ag-NPs was found at three steps/different decomposition temperatures of 166, 260, and 366 °C, with decomposition rates of − 2.4, − 3.0, and − 1.76, respectively, as seen in Fig. 9B.This finding demonstrates that the prepared Ag-NPs possessed high thermal stability and were capped with the SPS-phenolic compounds, which need three different temperatures to decompose.

### Antioxidant properties

The antioxidant capacity of the prepared SPS-Ag-NPs and SPS-phenolic extract was evaluated using DPPH assay and Trolox as an antioxidant standard. As seen in Table 4, the prepared SPS-Ag-NPs recorded significantly higher total antioxidant activity (13.8 µmol TE with a retention % of 115) than the SPS-phenolic extract (12 µmol TE) although the total phenolic content of the SPS extract (5 mg GAE) was higher than the phenolic content of the biosynthesized Ag-NPs (4.52 mg GAE). The results demonstrated that the potent antioxidant activity of the SPS-phenolic extract acted as a reducing agent and was used to synthesize Ag-NPs. In addition, the efficient antioxidant properties of the biosynthesized Ag-NPs can be attributed to the additive impact of the Ag-NPs on the SPS-phenolic compounds that were adsorbed over the spherically shaped Ag-NPs. Moreover, the antioxidant activity of the plant extract may be the most possible mechanism that assists in reducing the toxicity and adverse impacts of Ag-NPs^48,49^. Several studies showed enhanced/increased antioxidant activity of the biosynthesized Ag-NPs compared to the plant extracts employed in the biosynthesis process^41,48–51^.

**Table 4:** Total phenolic content and total antioxidant activity of the best-biosynthesized SPS-Ag-NPs compared to the SPS-phenolic extract.

Sample	TPC (mg GAE)	TPC retention %	TAA (µmol TE) using DPPH	TAA retention %
SPS-phenolic extract	5.0 ± 0.1^a^	100^a^	12.0 ± 0.4^a^	100^a^
SPS-Ag-NPs	4.52 ± 0.2^b^	90.4^b^	13.8 ± 0.3^b^	115^b^

TPC: Total phenolic content; TAA: Total antioxidant activity; GAE: Gallic acid equivalent; TE: Trolox equivalent; SPS: Saw palmetto seed phenolic extract; SPS-Ag-NPs: Sliver nanoparticles biosynthesized using saw palmetto seed phenolic extract. Results are presented as means ± SD (n = 3); results in the same column with different superscripts are significantly different at (*p* < 0.01).

### Antibacterial properties

The antibacterial activity of the biosynthesized SPS-Ag-NPs in comparison to the SPS-phenolic extract against multidrug-resistant human-enteric pathogenic bacteria, including Gram-negative *E. coli* and Gram-positive *S. aureus*, was evaluated. The biosynthesized Ag-NPs exhibited noticeably better antibacterial activity against Gram-negative *E. coli* and Gram-positive *S. aureus* with larger zones of inhibition (28.0 and 23.0 mm, respectively) compared to the SPS-phenolic extract (13.2 and 9.4 mm, respectively) and Amoxicillin (15.3–14.2 mm, respectively), as displayed in Table 5. Further, the biosynthesized Ag-NPs showed noticeably reduced MIC levels of 0.35 and 0.5 mg/ml against Gram-negative *E. coli* and Gram-positive *S. aureus*, respectively, compared to the SPS-phenolic extract (2.2 and 2.5 mg/ml, respectively) and Amoxicillin (2.0–2.1 mg/ml, respectively) (Table 5). The green-synthesis process of Ag-NPs improved/enhanced the antibacterial properties of the SPS-phenolic extract several folds. This high/enhanced antibacterial activity is due to the release of silver nanoparticles from the prepared Ag-NPs that serve as stores for these nanoparticles in addition to the effect of SPS-phenolic compounds that capped the Ag-NPs. These combined/multiple effects change bacteria’s structure and increase their membrane permeability^52,53^. In addition, the very small size of the Ag-NPs can adhere/enter the bacterial cell wall and bind to the bacterial proteins, resulting in bacterial DNA destruction, preventing bacterial proliferation/growth, and conclusively leading to bacterial death^54,55^. According to the results, the biosynthesized Ag-NPs were more effective against Gram-negative *E. coli* bacteria than Gram-positive *S. aureus* bacteria. This finding might be explained by the Gram-negative bacteria having a thin peptidoglycan layer, so Ag-NPs can easily penetrate it. In contrast, the Gram-positive bacteria have a thick layer of peptidoglycan, making the bacterial structure more rigid and resulting in the Ag-NPs penetrating more slowly than Gram-negative bacteria^56^.

**Table 5:** Antibacterial activity of the best-prepared SPS-Ag-NPs and the SPS-phenolic extract. Amoxicillin was used as a positive control.

Sample	Bacterial strain	
	*S. aureus*	*E. coli*
	Inhibition zone diameters (mm)	
SPS-phenolic extract	9.4 ± 0.4^a^	13.2 ± 0.62^a^
Biosynthesized SPS-Ag-NPs	23 ± 1.1^b^	28 ± 0.87^b^
Amoxicillin	14.2 ± 0.71^c^	15.3 ± 0.82^c^
	MIC (mg/ml)	
SPS-phenolic extract	2.5 ± 0.12^a^	2.2 ± 0.11^a^
Biosynthesized SPS-Ag-NPs	0.50 ± 0.03^b^	0.35 ± 0.02^b^
Amoxicillin	2.1 ± 0.10^c^	2.0 ± 0.11^c^

SPS: Saw palmetto seed phenolic extract; SPS-Ag-NPs: Sliver nanoparticles biosynthesized using saw palmetto seed phenolic extract. Results are presented as means ± SD (n = 3); results in the same column with different superscripts are significantly different at (*p* < 0.01).

## Conclusion

This study described a simple, inexpensive, and environmentally friendly biosynthesis method for producing Ag-NPs using SPS-phenolic extract, which contains high amounts of phenolic acids with potent antioxidant activity. A statistical approach and sixteen experiments were executed to determine the ideal conditions of the biosynthesis process for achieving the maximum yield of Ag-NPs with the highest phenolic content. The best-obtained SPS-Ag-NPs displayed spherical shapes by TEM images with sizes ranging from 11.17 to 38.32 nm and great stability via a high negative ZP value. The FTIR-spectra demonstrated that SPS-phenolic compound functional groups are implicated in sliver bio-reduction and in stabilizing, capping, and preventing Ag-NPs from aggregation. According to the thermogravimetric study, the prepared Ag-NPs have great thermal stability and require three different temperatures to decompose. The prepared SPS-Ag-NPs exhibited potent antioxidant and antibacterial activity compared to the SPS extract. In light of these findings, this study recommends large commercial production of these optimized SPS-Ag-NPs as a potent biomedical tool and an antibacterial agent.

## Data Availability

The datasets generated during and/or analyzed during the current study are available from the corresponding author upon reasonable request.

## Abbreviations

DPPH: 1,1-Diphenyl-2-picrylhydrazyl
CCD: Central composite design
DTG: Differential thermogravimetric
DLS: Dynamic light scattering
FT-IR: Fourier transform infrared
GAE: Gallic acid equivalent
MIC: Minimum inhibition concentration
NPs: Nanoparticles
RSM: Response surface methodology
SPS: Saw palmetto seed
SPS-phenolic extract: Saw palmetto seed phenolic extract
Ag-NPs: Silver nanoparticles
SPS-Ag-NPs: Sliver nanoparticles biosynthesized using saw palmetto seed phenolic extract
SPR: Surface plasmon resonance
TGA: Thermogravimetric analysis
TAA: Total antioxidant activity
TPC: Total phenolic content
TEM: Transmission electron microscopy
TE: Trolox equivalent
ZP: Zeta potential
